# Recommendations for whole genome sequencing in diagnostics for rare diseases

**DOI:** 10.1038/s41431-022-01113-x

**Published:** 2022-05-16

**Authors:** Erika Souche, Sergi Beltran, Erwin Brosens, John W. Belmont, Magdalena Fossum, Olaf Riess, Christian Gilissen, Amin Ardeshirdavani, Gunnar Houge, Marielle van Gijn, Jill Clayton-Smith, Matthis Synofzik, Nicole de Leeuw, Zandra C. Deans, Yasemin Dincer, Sebastian H. Eck, Saskia van der Crabben, Meena Balasubramanian, Holm Graessner, Marc Sturm, Helen Firth, Alessandra Ferlini, Rima Nabbout, Elfride De Baere, Thomas Liehr, Milan Macek, Gert Matthijs, Hans Scheffer, Peter Bauer, Helger G. Yntema, Marjan M. Weiss

**Affiliations:** 1 Center for Human Genetics, KU Leuven, Gasthuisberg, Laboratory for Molecular Diagnosis, Leuven, Belgium; 2 CNAG-CRG, Centre for Genomic Regulation (CRG), The Barcelona Institute of Science and Technology, Barcelona, Spain; 3 Universitat Pompeu Fabra (UPF), Barcelona, Spain; 4 Departament de Genètica, Microbiologia i Estadística, Facultat de Biologia, Universitat de Barcelona (UB), Barcelona, Spain; 5 Erasmus MC University Medical Center - Sophia Children’s Hospital, Department of Clinical Genetics, Rotterdam, The Netherlands; 6 Illumina, Inc., Department of Molecular and Human Genetics, Baylor College of Medicine, Houston, TX USA; 7 Dept of Pediatric Surgery, Rigshospitalet, Faculty of Health and Medical Sciences, Copenhagen University, Denmark, Dept. of Women’s and Children’s health, Karolinska Institute, Stockholm, Sweden; 8 Institute of Medical Genetics and Applied Genomics, University of Tübingen, Tübingen, Germany; 9 Department of Human Genetics and Radboud Institute for Molecular Life Sciences, Radboud University Medical Centre, Nijmegen, 6525 GA The Netherlands; 10 Agilent Technologies, Diagnostics and Genomics Group, Leuven, Belgium; 11 Department of Medical Genetics, Haukeland University Hospital, 5021 Bergen, Norway; 12 Department of Genetics, University Medical Center Groningen, University Groningen, Groningen, The Netherlands; 13 Manchester Centre For Genomic Medicine, University of Manchester, St Mary’s Hospital, Manchester, M13 9WL UK; 14 Division of Evolution and Genomic Sciences School of Biological Sciences University of Manchester, Manchester, UK; 15 Hertie-Institute for Clinical Brain Research, University of Tübingen, Tübingen, Germany; 16 German Center for Neurodegenerative Diseases (DZNE), Tübingen, Germany; 17 Department of Human Genetics, and Donders Centre for Cognitive Neuroscience, Radboud University Medical Center, Nijmegen, The Netherlands; 18 Genomics Quality Assessment, NHS Lothian, Edinburgh, Scotland; 19 Lehrstuhl für Sozialpädiatrie, Technische Universität München, Munich, Germany; 20 Zentrum für Humangenetik und Laboratoriumsdiagnostik (MVZ), Martinsried, Germany; 21 MVZ Martinsried GmbH, Martinsried, Germany; 22 Amsterdam University Medical Centers, location AMC, Department of Clinical Genetics, Amsterdam, The Netherlands; 23 Highly Specialised Osteogenesis Imperfecta Service and Sheffield Clinical Genetics Service, Sheffield Children’s NHS Foundation Trust, Sheffield, UK; 24 Department of Oncology & Metabolism, University of Sheffield, Sheffield, UK; 25 University Hospital Tübingen, Institute for Medical Genetics and Applied Genomics and Centre for Rare Diseases, Calwerstr. 7, 72076 Tübingen, Germany; 26 Dept of Clinical Genetics, Box 134, Cambridge University Hospitals, Cambridge, UK; 27 Unit of Medical Genetics, University Hospital & Department of Medical Sciences, University of Ferrara, Ferrara, Italy; 28 Pediatric Neurology. reference centre for rare epilepsies. Hôpital Necker Enfants malades, APHP, Université de Paris, Institut Imagine (INSERM UMR 1163), Paris, France; 29 Department of Biomolecular Medicine, Ghent University, Ghent, Belgium; 30 Center for Medical Genetics, Ghent University Hospital, Ghent, Belgium; 31 Jena University Hospital, Friedrich Schiller University, Institute of Human Genetics, Jena, Germany; 32 Department of biology and medical genetics, 2nd Faculty of Medicine Charles University and University hospital Motol, Prague, Czechia; 33 Radboud university medical center, Department of Human Genetics, P.O. Box 9101, 6500 HB Nijmegen, The Netherlands; 34 CENTOGENE GmbH, Am Strande 7, 18055 Rostock, Germany

**Keywords:** Next-generation sequencing, Medical genetics

## Abstract

In 2016, guidelines for diagnostic Next Generation Sequencing (NGS) have been published by EuroGentest in order to assist laboratories in the implementation and accreditation of NGS in a diagnostic setting. These guidelines mainly focused on Whole Exome Sequencing (WES) and targeted (gene panels) sequencing detecting small germline variants (Single Nucleotide Variants (SNVs) and insertions/deletions (indels)). Since then, Whole Genome Sequencing (WGS) has been increasingly introduced in the diagnosis of rare diseases as WGS allows the simultaneous detection of SNVs, Structural Variants (SVs) and other types of variants such as repeat expansions. The use of WGS in diagnostics warrants the re-evaluation and update of previously published guidelines. This work was jointly initiated by EuroGentest and the Horizon2020 project Solve-RD. Statements from the 2016 guidelines have been reviewed in the context of WGS and updated where necessary. The aim of these recommendations is primarily to list the points to consider for clinical (laboratory) geneticists, bioinformaticians, and (non-)geneticists, to provide technical advice, aid clinical decision-making and the reporting of the results.

## Introduction

EuroGentest is a European initiative, aiming to promoting accurate, reliable and high-quality genetic diagnostics across Europe. Initially funded by the European commission, EuroGentest has been integrated in the European Society of Human Genetics (ESHG) as a working group. In 2016, EuroGentest published guidelines, endorsed by the ESHG, for diagnostic Next Generation Sequencing (NGS) applications for rare genetic diseases [1]. These previous recommendations focused on Whole Exome Sequencing (WES) and targeted (gene panels) sequencing detecting small germline variants (Single Nucleotide Variants (SNVs) and insertions/deletions (indels)). They consisted of 38 statements dealing with different aspects of diagnostic genome-wide analysis.

The exome represents only about 1–2% of the entire genome and therefore WES requires specific enrichment methods to amplify the exome for sequencing. This is often limited to approximately 99% of the exome being enriched, and this uneven coverage may compromise Copy Number Variant (CNV) detection. Also, other Structural Variants (SVs) like inversions or variants in regulatory or intronic regions are usually missed in WES diagnostics. Taking into account these limitations, across all disease entities, the underlying disease variant can be detected in 5–50% of all patients [2–5]. WGS in principle allows the detection of disease relevant genomic variants beyond the exome such as DNA structural alterations, deep intronic variants, variants in non-coding regions, or repeat expansions [6, 7], and may improve variant calling in homologous sequences [8]. It is beyond the scope of this paper to make a full comparison of the diagnostic utility of targeted sequencing verses WGS. Each laboratory has to consider which technologies are the most appropriate for the diagnostic tests they are offering.

Diagnostic NGS initially was developed with a focus on disease-associated gene panels based on exon enrichment. In this respect, diagnostic WGS does not compete with or substitute exon-based sequencing. Rather it represents a novel, distinct diagnostic tool that targets genes and goes beyond the coding region and allows elucidation of established and novel non-coding genomic diseases. This notion implies that any gene panel, i.e. target region definition stemming from exon-based enrichment technology, needs careful revision regarding the targeted regions. Therefore, the scope of the addressed reportable range, constitutionally, has to go beyond our current standards and include in addition to the coding sequence, at least all known and/or interpretable (validated) non-coding regulatory and splicing-relevant regions, i.e. the full genomic sequence from 5-prime through 3-prime UTR and eventually beyond. Moreover, disease-associated non-coding genomic regions (for example *D4Z4* for muscular dystrophies or the trinucleotide repeat in the 5-prime UTR of the *FMR1* gene for intellectual disability) have to be accounted for in phenotype-related target region definition and therefore ‘phenotype-related gene panels’ are not sufficient in diagnostic WGS.

Hence, the use of WGS in diagnostics warrants evaluating and updating of the 2016 NGS guidelines. All 38 statements from the previous guidelines [1] have been reviewed in the context of WGS and updated where necessary (see supplementary table 1). One of the 38 statements, statement 07 on a simple rating system, is not discussed as it cannot be applied to WGS given the different type of variants that can be detected (see discussion on reportable range). The updated recommendations now consist of a combination of 44 original, updated and new statements. Only new and updated statements are listed in the present text and discussed in supplementary information 1.

This report was jointly initiated by EuroGentest and the Horizon2020 project Solve-RD. Solve-RD has the ambition to elucidate the genetic cause of the majority of currently unsolved rare genetic disorders by a variety of analytical techniques. WGS and uniform clinical and genomic data-analysis are central in this project. To provide the link to reliable clinical application of the obtained information, EuroGentest, as a work package leader in the project, has the task to update and produce diagnostic WGS recommendations.

The task has been undertaken by organizing expert meetings in February 2019 and September 2019. Colleagues with different fields of expertise and backgrounds from different countries across Europe and beyond were involved, as well as representatives of the European Reference Networks (ERNs, https://ec.europa.eu/health/ern_en). ERNs are virtual networks involving healthcare providers across Europe. They aim to tackle complex or rare diseases and conditions that require highly specialized treatment and a concentration of knowledge and resources. The following ERNs were represented: genetic tumor risk syndromes (GENTURIS), congenital malformations and rare intellectual disability (ITHACA), neuromuscular diseases (NMD), neurological diseases (RND), immunodeficiency, autoinflammatory and autoimmune diseases (RITA), urogenital diseases and conditions (UROGEN), eye diseases (EYE), inherited and congenital anomalies (ERNICA), rare neurological diseases (BOND) and rare and complex epilepsies (EpiCARE).

This report has been finalized in May 2021 and endorsed by the Solve-RD Steering Committee, the representing ERNs, the European Board of Medical Genetics (EBMG) and the ESHG.

The recommendations focus on diagnostic NGS sequencing including WGS in a clinical setting for rare disease diagnostics, although most of the statements also apply to the identification of somatic variants in cancer diagnostics. Clearly, an evaluation of the limit-of-detection (LoD) is not typically performed when NGS is used to identify constitutional variants, but this is an essential requirement for somatic testing. An evaluation of LoD requires a dilution series of a characterized sample and is essential for determining the appropriate sequencing depth and for the validation of the bioinformatic pipelines. The aim of moving to WGS is to be able to simultaneously detect CNVs and chromosomal anomalies, as well as SNVs for monogenic and oligogenic diseases and cancers. Applications of the different NGS approaches to multifactorial disorders and pharmacogenomics are not included. The use of tools to determine polygenic risks scores (PRS) and to calculate relative risks on the basis of association studies, is not covered in these recommendations.

The recommendations cover aspects from the evaluation and rationale to set up diagnostic NGS applications, including quality control of the different aspects of the laboratory (wet work) procedure and bioinformatics pipelines, variant interpretation and data banking, to reporting of NGS results. The use of WGS for research is not addressed specifically, but quality rules will equally apply to such analysis. The requisites for providing NGS diagnostics are of course its clinical utility, the use of state-of-art sequencing technologies, diagnostic routing (i.e. routing of genetic tests within the laboratory for a specific disease) [9] and variant analysis, and the generation of reports in a diagnostic setting.

## Glossary and definitions

ACMG: American College of Medical Genetics (https://www.acmg.net/)

CNV: copy number variant

ClinGen:https://clinicalgenome.org/

ClinVar: https://www.ncbi.nlm.nih.gov/clinvar/

DECIPHER: https://deciphergenomics.org

CPMS: Clinical Patient Management System: (https://ern-euro-nmd.eu/clinical-patient-management-system/)

EBMG: European Board of Medical Genetics (https://www.ebmg.eu)

ERN: European Reference Network: (https://ec.europa.eu/health/ern_en)

ESHG: European Society of Human Genetics (https://www.eshg.org/)

EQA: External Quality Assessment

GenCC: The Gene Curation Coalition (https://thegencc.org/)

GA4GH: Global Alliance for Genomics and Health (https://www.ga4gh.org)

GDPR: General Data Protection Regulation

GIAB: Genome in a bottle (https://www.nist.gov/programs-projects/genome-bottle)

gnomAD:Genome Aggregation Database https://gnomad.broadinstitute.org/

HPO: human phenotype ontology (https://hpo.jax.org/app/)

INDEL: insertion deletion

LoD: limit-of-detection

MLPA: Multiplex Ligation-dependent Probe Amplification

mtDNA: mitochondrial DNA

NGS: next-generation sequencing

OMIM: Mendelian Inheritance in Man (https://www.omim.org/)

PanelApp: https://panelapp.genomicsengland.co.uk/

PRS: polygenic risk scores

RD: rare disease

SNV: single nucleotide variant

SV: structural variant

UF:unsolicited finding

UPD: uniparental disomy

VUS: variants of uncertain significance

WES: whole exome sequencing

WGS: whole genome sequencing

### General recommendations

The WGS technology and applications are constantly changing and are still improving. This should not prevent the implementation of WGS in diagnostics as WGS offers a potential overall benefit for the patient. However, before implementation in a clinical diagnostic setting, the test needs to be sufficiently validated.

- RECOMMENDATION 1: It is recommended to introduce WGS analysis in a diagnostic setting when it is a relevant improvement on quality, efficiency and/or diagnostic yield.
- RECOMMENDATION 2: Diagnostic WGS for rare diseases and cancer (as well as other genetic testing approaches) should only be performed in accredited laboratories.
- RECOMMENDATION 3: NGS should not be transferred to clinical practice without acceptable validation of the tests.
- RECOMMENDATION 4: Confirmation, interpretation and communication to the patient of results obtained in a research setting should always be done after re-testing on (preferably) an independent sample by a diagnostic laboratory.

### Diagnostic routing

In general, the purpose of a diagnostic route is to choose the most efficient and relevant diagnostic strategy to reach a molecular diagnosis (both in time and costs). Although WGS (and WES) are increasingly being implemented as a first-tier diagnostic test, referring clinicians should be aware that there might be more efficient diagnostic tests for specific diseases. Diagnostic routing describes the flow of samples and available genetic tests for a given disease in a flowchart and can provide insight into the diagnostic processes and possibilities [9]. A diagnostic route may contain different laboratory techniques (e.g., multiplex ligation-dependent probe amplification (MLPA), Sanger sequencing, and NGS), and also target different genes. In this context, classical genetic tests may precede WGS and other NGS applications. Diagnostic routes in which specific genes could be analyzed before WGS have mainly to do with the high mutation rate in these genes for a specific disorder, e.g., *FBN1* in classical Marfan syndrome, *SCN1A* for Dravet/SMEI syndrome or *CFTR* for cystic fibrosis.

- RECOMMENDATION 5: The laboratory should provide information to the clinician for which type of variants the genetic test is validated.
- RECOMMENDATION 6: Limitations of WGS should be considered and communicated to the referring clinician.
- RECOMMENDATION 7: For diagnostic purposes only genes for which a clear association with the disease has been confirmed, should be reported. Variants in genes of unknown function may be listed in an independent research report.
- RECOMMENDATION 8: Diagnostic testing should be directed towards answering the clinical question. It is recommended to preferably analyze one (or more) in silico gene panels and use filtering strategies, and, use trios for disorders frequently caused by de novo variants.
- RECOMMENDATION 9: For the interpretation of variants in genes causing a monogenic disorder the ‘5 tier classification system’ should be used.
- RECOMMENDATION 10: Large CNVs should be interpreted using databases including cytogenomic aberrations.
- RECOMMENDATION 11: It is recommended to analyze and report variants outside the exome only when they are (likely) pathogenic. VUS shall (only) be reported in case follow up studies can provide more insight into pathogenicity.
- RECOMMENDATION 12: For interpretation of the variants, it is necessary to have clinical information of the patient (and the parents when trio analysis is performed), preferably in standardized terms, such as HPO.
- RECOMMENDATION 13: The diagnostic laboratory has to implement/use a structured database for all classified variants with current annotations.
- RECOMMENDATION 14: Reported variants should be shared by submitting them to federated, regional, national, and/or international databases, accessible by laboratory geneticists and researchers.

### Bioinformatics

The following section addresses points to consider when implementing and validating the bioinformatics pipeline used to analyse WGS data. The bioinformatics pipeline is defined as all software used from raw data analysis to variant annotation/prioritisation. Recommendations are also given on the data formats, storage and validation procedures.

- RECOMMENDATION 15: The use of the most recent annotated reference genome version is recommended.
- RECOMMENDATION 16: Standard data formats should be used.
- RECOMMENDATION 17: The bioinformatics pipeline must be tailored for the technical platform used.
- RECOMMENDATION 18: It is recommended to develop and define a protocol to keep the bioinformatics tools used for variant calling and variant annotation up to date.
- RECOMMENDATION 19: Optimally characterized reference samples should be used for the validation and standardization of bioinformatics tools.
- RECOMMENDATION 20: The diagnostic laboratory has to validate all parts of the bioinformatics pipeline (public domain tools or commercial software packages) with standard data sets periodically and whenever relevant changes (new releases) are implemented.
- RECOMMENDATION 21: Quality parameters to monitor the analytical process (in process controls) and to measure performance of the used techniques should be adopted. For coding regions, general data quality should be at least similar to that from WES data. All NGS quality metrics used in diagnostics procedures should be accurately described and, ideally, stored in a database.
- RECOMMENDATION 22: The bioinformatics pipeline should be validated for all reportable types of variants, minimally including SNVs, small indels, and CNVs.
- RECOMMENDATION 23: All WGS variants should be annotated.
- RECOMMENDATION 24: It is recommended to record variant frequencies in an in-house database.
- RECOMMENDATION 25: The diagnostic laboratory should implement a protocol for long-term storage of all relevant data sets.

### Quality assessment

Statements about WGS validation and quality assessment are given in the following section. In house validation and comparison of test performance is intended to ensure a product results in an outcome (or portion thereof, or set thereof) that meets the operational needs of the user. It is about the performance and use of a test/method and it should not be confused with validation/confirmation of (the presence of) a variant. Most aspects of validation and verification that are included in the Matthijs, Souche *et al*. paper from 2016 are still applicable [1].

- RECOMMENDATION 26: The reportable range, that is, the portion of the clinical target for which reliable calls can be generated, has to be defined during the test development and should be available to the clinician.
- RECOMMENDATION 27: If DNA from different tissue types (*e.g.*, blood and saliva) is tested diagnostically, each tissue type should be validated separately for both wet and dry laboratory procedures.
- RECOMMENDATION 28: Whenever major changes are made to the test, quality parameters have to be checked, and a set of validation samples has to be re-run as part of the validation.
- RECOMMENDATION 29: Aspects of sample tracking and the installation of barcoding to identify samples should be dealt with during the evaluation of the assay and included in the platform validation.
- RECOMMENDATION 30: Variants compliant with predefined quality metrics do not require confirmation by a second technique.

### Ethical considerations

The implementation of WGS has increased the probability of detecting variants predisposing to a disease other than the initial clinical question (unsolicited or incidental findings; UFs or IFs). For example, one will be able to detect diseases like Huntington disease (provided that a laboratory is able to detect repeat expansions). Although the risk and the type of UF are different for WGS, the statements concerning information for requesting clinicians are essentially unaltered. The discussion of the benefits and harms of returning UFs are beyond the scope of this paper, as well as the intended search for disease risks other than the initial request (secondary findings).

- RECOMMENDATION 31: Laboratories should have a clearly defined protocol for addressing unsolicited findings prior to launching the test.
- RECOMMENDATION 32: Clinicians should provide genetic counseling and obtain informed consent prior to clinical WGS.
- RECOMMENDATION 33: The laboratory should anticipate possible follow up studies resulting from the dissemination of unsolicited findings.
- RECOMMENDATION 34: The laboratory is not expected to re-analyze data systematically and report novel findings, unless explicitly requested to do so or for quality assurance activity.
- RECOMMENDATION 35: The results of a diagnostic test, particularly by analysis of a whole genome, might not be conclusive but may be hypothesis generating.
- RECOMMENDATION 36: WGS data can only be used for research purposes with adequate informed consent.

### Reporting

The genetic test report should provide a clear, concise, accurate, fully interpretative and authoritative answer to the clinical question [10, 11].

- RECOMMENDATION 37: For each NGS test, the laboratory has to provide the following: the diagnostic strategy, the types of genetic variants detected, their reportable range, the analytical sensitivity and precision.
- RECOMMENDATION 38: The report of an NGS assay should summarize the patient’s identification and reason for referral, a brief description of the test, a summary of results, and the major findings on one page.
- RECOMMENDATION 39: Both the reference genome build and, when applicable, the gene reference transcript version should be mentioned in each report.
- STATEMENT 40: An OMIM reference should be reported where available (https://www.omim.org/).
- RECOMMENDATION 41: A local policy, in line with international recommendations, for reporting genomic variants should be established and documented by the laboratory prior to providing analysis of this type.
- RECOMMENDATION 42: VUS should be reported only if the phenotype associated with the respective (disease) gene matches with the clinical features of the patient and when follow up studies can be performed to gain more information about pathogenicity of the variant.
- RECOMMENDATION 43: Exploratory findings that are beyond the confirmation or exclusion of a clinical diagnosis should be reported.
- RECOMMENDATION 44: WGS reports should be delivered to the referring physician. Advice to refer the patient and family for genetic counselling must be included in the report.

### Perspectives

As the scope of a diagnostic offering has considerably grown with the implementation of WGS, it should not be considered as just as “the better exome sequencing backbone”. A full diagnostic analysis and interpretation of all known genomic alterations associated with a given phenotype should be performed. In this respect, we suggest to use the term “gene panel sequencing” for traditional coding sequence centered approaches and use *“phenotype-related genomic regions”* as a term to define target regions in diagnostic WGS.

Furthermore, in these recommendations, we intentionally did not focus on the interpretation of mitochondrial DNA (mtDNA) variants, or somatic variants in WGS data, mainly because of technical limitations of the current WGS technology. For example, somatic variants challenge the economic ability to produce deeper coverage datasets, which is currently not realistic at scale. On a similar note, developing and deploying tools for HLA genotyping and mtDNA interpretation will rely on phasing and haplotyping, both being recognized as a limitation of short-read sequencing. Another limitation of short-read sequencing technology is that it does not directly capture epigenetic DNA modification and thereby markers of related epigenetic phenotypes. This part of the diagnostic spectrum is not specifically addressed in these recommendations. However, these DNA markers of epigenetic phenotypes are considered relevant and should be accounted for in the diagnostic routing.

Going forward, technologies for long-read (single molecule) sequencing and DNA modification identification will be foreseeable add-ons to our diagnostic toolbox. This will require to develop additional diagnostic (software) tools to increase the resolution, utility, and value of diagnostic WGS. Consequently, any contribution in these areas is highly welcome for the updated version of these recommendations to come.

## Supplementary information

All supplementary_Information
