# Supplementary material for: Recommendations for whole genome sequencing in diagnostics for rare diseases
Source: Eur J Hum Genet. 2022 May 16;30(9):1017–21. doi: 10.1038/s41431-022-01113-x (PMC9437083; doi:10.1038/s41431-022-01113-x)
Supplement: Supplementary file 1 — All supplementary_Information [file 41431_2022_1113_MOESM1_ESM.pdf]

Supplementary Information

Supplementary information 1: Extended guidelines.

All WGS statements are further outlined.

Supplementary table 1: WGS statements and link to WES statements.

Supplementary table 2: List of WGS processing steps, bioinformatics tools, databases and file formats.

All the elements that should be part of the NGS analysis pipeline are described in table S1 as well as examples of software that can be used and output formats.

Supplementary table 3: Metrics that can be used for WGS quality control.

Tracking QC metrics throughout the whole analysis pipeline is essential to ensure that each final report is based on diagnostics-grade read data. Commonly used quality metrics are outlined this table.

Supplementary information 2: Impact of library quality and coverage on sensitivity and precision.

Assessment of library quality and sequencing depth on WGS quality and performance.

Supplementary information 1: Extended guidelines.

General recommendations

- **RECOMMENDATION 1: It is recommended to introduce WGS analysis in a diagnostic setting when it is a relevant improvement on quality, efficiency and/or diagnostic yield.**

  WES and gene panels have been commonly used in a diagnostic setting for many years. WGS can be recommended based on increased quality and diagnostic yield. For instance, WGS allows the detection of SNVs and CNVs outside the exome (1-3), and provides a better coverage of coding regions than WES (4).

  It is recommended to use WGS, even if only the exome or a gene panel are bioinformatically extracted from the genome (in silico). Extra yield will be expected, e.g. in GC rich regions. The extra sequencing costs may be compensated by the fact that (in the near future) fewer additional tests will be required, such as separate analysis of tandem repeats, single exon deletions and paralogous or repetitive sequences, as soon as mapping issues for these applications have been resolved, either by long-read sequencing or by bioinformatics mapping solutions (5, 6).

- **RECOMMENDATION 2: Diagnostic WGS for rare diseases and cancer (as well as other genetic testing approaches) should only be performed in accredited laboratories**, *e.g. for medical laboratories in Europe complying with the ISO15189:2012 standard (most recent version at the time of writing this manuscript) and/or within National Health System fully accredited laboratories with equivalent accreditation modalities. WGS must be validated and incorporated in the scope of the accredited laboratory, and the laboratory should successfully participate in external quality assessment (EQA) schemes. This guarantees that a framework*

of quality management is in place, and that tests are performed by qualified, competent staff. Interpretation and reporting should be performed by experienced molecular geneticists (e.g. registered clinical laboratory geneticists). The European board of medical genetics (EBMG) has developed a set of standards and a curriculum for clinical laboratory geneticists undertaking analysis of genetic data. It is recommended to use these standards for the specialists that are responsible for the interpretation of the variants (https://www.ebmg.eu) and to include NGS testing in the training.

- **RECOMMENDATION 3: NGS should not be transferred to clinical practice without acceptable validation of the tests**.

  More details on NGS and especially WGS validation can be found in recommendations 16, 17, 18, 19, 20, 21, 22 in the bioinformatics section and recommendations 26 and 27 in the quality assessment sections.

- **RECOMMENDATION 4: Confirmation, interpretation and communication to the patient of results obtained in a research setting should always be done after re-testing on (preferably) an independent sample by a diagnostic laboratory.**

  The referring clinician should connect with a diagnostic laboratory to confirm any research results relevant to the phenotype. This is important to maintain the quality standards for clinical testing, to warrant that the results of a genetic test are recorded in the appropriate place in the patient's health files, and to keep the lines of communication and standards of variant interpretation clear and consequent. For this reason, the clinical laboratory should be ISO15189-accredited, or equivalent (see recommendation 2).

Diagnostic routing

- **RECOMMENDATION 5: The laboratory should provide information to the clinician for which type of variants the genetic test is validated.**

  With WGS analysis it is theoretically possible to detect more types of variants than within an exome, for example, repeat expansions, CNVs, inversions and translocations. As part of the validation, the sensitivity and specificity should be estimated and stated per type of variant. This might have complications for the diagnostic yield of a specific phenotype (see limitations at recommendation 6).

- **RECOMMENDATION 6: Limitations of WGS should be considered and communicated to the referring clinician.**
  - *With short-read sequencing technology, some regions remain challenging because of e.g. high GC content.*
  - *Detection of mosaicisms is not always possible, since this is related to the percentage of mosaicism, the variant type and the coverage depth.*
  - *Short-read sequencing technology allows the detection of repeat expansions. However, the accurate size estimation remains challenging (7). Testing for repeat expansions if clinically suggestive is recommended as a gene-specific, complementary analysis to WGS.*
  - *Structural variations (SVs) are also difficult to detect with short-read sequencing. Sensitivity to the detection of SVs may be improved using long-read sequencing.*
  - *Epigenetic causes of disease are missed unless specific methods are applied.*

- **RECOMMENDATION 7**: **For diagnostic purposes only genes for which a clear association with the disease has been confirmed, should be reported. Variants in genes of unknown function may be listed in an independent research report.**

  It is recommended to use gene lists to facilitate analysis.

  - ***Definition of gene lists:*** *For the creation and curation of gene lists, the use of key resources like the Gene Curation Coalition (GenCC, https://thegencc.org/), ClinGen (https://search.clinicalgenome.org/kb/gene-validity), OMIM (https://www.omim.org) and DECIPHER (https://www.deciphergenomics.org/ddd/ddgenes) is recommended.*
    - ***Candidate genes:*** *it is recommended not to include candidate genes (genes that have not yet been linked to disease but elect as candidates for disease e.g. on the basis of a known function in a specific cellular pathway) in gene panels, because they might lead to inconclusive and possible confusing situations for both clinician and patient.*
    - ***Core genes*** *are disease genes that are considered essential to establish a molecular diagnosis as they have significant pathogenic variant frequencies for that particular genetic disease. WGS should allow very high-quality genotyping of coding and known pathogenic noncoding variants. It is mandatory that CNV analysis is included for gene dosage associated diseases.*
  - ***Management of gene lists:*** *For in silico panel analysis, tools such as PanelApp from Genomics England and managed lists provided by European Reference Networks (ERNs) (1) are recommended. In PanelApp all panels are named, versioned and updated. PanelApp has 3 categories; green, amber and red. The categories green and amber have enough evidence to be included in gene lists while it is recommended to exclude the category red from diagnostic gene panels (https://panelapp.genomicsengland.co.uk/#!Guidelines).*

- **RECOMMENDATION 8: Diagnostic testing should be directed towards answering the clinical question. It is recommended to preferably analyze one (or more) *in silico* gene panels and use filtering strategies, and, use trios for disorders frequently caused by *de novo* variants.**
  - *It is recommended to only search for pathogenic variants in genes associated with the phenotype of the patient. The option for analyzing other genes for secondary findings depends on local policy [ESHG policy (8), American College of Medical Genetics (ACMG) (9)].*
  - *When no gene panel exists the entire exome can be analyzed, and when the phenotype is known to be associated with other types of genomic aberrations (such as CNVs, repeat expansions), the appropriate test should be offered (first) (see diagnostic routing).*
  - *It may still be preferable not to analyse the entire exome but restrict the analysis to all genes proven to cause a monogenic disorder and validated for clinical diagnostics (referred to as 'clinical exome' or 'mendeliome').*
  - *Filtering strategies may include inheritance filtering (e.g. for de novo variants, homozygosity), and allele frequency filtering. Phenotype prioritization (e.g. based on Human Phenotype Ontology (HPO) terms) may be of added value but should be used with caution (10). The diagnostic report has to state clearly which genes were included in the analysis (see recommendation 36).*

- **RECOMMENDATION 9: For the interpretation of variants in genes causing a monogenic disorder the '5 tier classification system' should be used.**

  The use of specific standard terminology - "pathogenic," "likely pathogenic," "uncertain significance," "likely benign," and "benign" - was originally developed for variants identified in genes that cause Mendelian disorders (11). In addition, the ACMG guidelines provide criteria for variant interpretation and several adaptations have been outlined for specific diseases

(https://clinicalgenome.org/working-groups/sequence-variant-interpretation/, https://cspec.genome.network/cspec/ui/svi/). Several alternatives of this classification system have been described (12-15). For non-Mendelian disorders this classification system should not be used.

- **RECOMMENDATION 10: Large CNVs should be interpreted using databases including cytogenomic aberrations.**

  Large CNVs (i.e. affecting multiple genes) should not be interpreted using the same strategies for small variants. Instead (molecular) cytogenetic oriented nomenclature (ISCN 2020 - ISBN 978-3-318-06706-4), corresponding databases ((16-18); CytoGenomics databases: http://cs-tl.de/DB.html) or simple literature search [PubMed or Google] should be used for interpretation and aligning with previously published comparable cases. Databases like DECIPHER, containing all types of genotypic data (e.g. SNVs, CNVs, translocations, UPD) are extremely valuable for the interpretation of WGS data. The possible underlying cytogenetic equivalents of the detected CNVs should be considered, including especially their different implications for inheritance (19) and correlation with imprinting (14).

- **RECOMMENDATION 11: It is recommended to analyze and report variants outside the exome only when they are (likely) pathogenic. VUS shall (only) be reported in case follow up studies can provide more insight into pathogenicity.**

  Laboratories can have a different definition of the exome, but it should at least include the consensus coding regions and splice sites. Examples of variants outside the exome are variants located in regulatory regions, intronic or conserved non-genic regions.

  VUS outside the exome should only be reported if either:

  - *sufficient evidence in scientific literature or confirmed data in databases exists for effect on regulation of expression, splicing, or other functional effects*

- *or an in-house functional test can be performed to provide enough evidence, e.g. aberrant transcript analysis*
- *or the intronic VUS is located in a gene matching the patient's phenotype, either in trans with an exonic (likely) pathogenic variant, or homozygous (for recessive disorders), or de novo, or ultra-rare.*

- **RECOMMENDATION 12: For interpretation of the variants, it is necessary to have clinical information of the patient (and the parents when trio analysis is performed), preferably in standardized terms, such as HPO.**

  To improve variant interpretation, efficient (and in real-time) communication between the laboratory and the clinician is crucial (15, 20). It is recommended that the clinical information is delivered in a standardized way, for example by using HPO (https://hpo.jax.org/app/). The genotype and phenotype should be in data formats that allow data sharing such as the Clinical Patient Management System (CPMS; https://ern-euro-nmd.eu/clinical-patient-management-system/) that the ERNs use for virtual multidisciplinary teams, or phenopackets (www.phenopackets.org), a file format for transmitting phenotype terms between health records, laboratories, and research database. Ideally, the system would be capable of using these terms in different languages. Negative criteria are often relevant (like normal head circumference and height, and lack of developmental delay or the lack of minor symptoms in unaffected parents).

- **RECOMMENDATION 13: The diagnostic laboratory has to implement/use a structured database for all classified variants with current annotations.**

  Such a database is necessary to monitor all classified variants and eventually reclassify VUS as soon as enough information is available to classify them as (likely) benign or (likely) pathogenic variants. This is a requisite for all laboratories that provide diagnostic testing: it has to be

possible to go back to previous patient records when variants are reclassified on the basis of novel knowledge. There is no obligation to continuously scrutinize literature and variant databases for changes in the status of individual variants.

- **RECOMMENDATION 14: Reported variants should be shared by submitting them to federated, regional, national, and/or international databases, accessible by laboratory geneticists and researchers.**

From a community standpoint, information on variants and the interpretation of their pathogenic nature, should be shared. There are different possibilities for sharing reported variants: for example, DECIPHER (https://deciphergenomics.org) (21), ClinVar (https://www.ncbi.nlm.nih.gov/clinvar/), dbVar (https://www.ncbi.nlm.nih.gov/dbvar/), eMERGE (https://emerge-network.org/) (22), LOVD ((https://www.lovd.nl/), WiNGS/NGS-Logistics (https://wings.esat.kuleuven.be) (23), VKGL database (Dutch initiative, https://www.molgenis.org/vkgl) (24). Besides the non-disease specific databases there are many disease- or gene specific databases (25). Please note that sharing of genotype and/or phenotype information should adhere to General Data Protection Regulations (GDPR).

- *To ensure that feedback regarding non-reported (non-actionable) variants will be gathered, we recommend to submit these variants and/or candidate genes in a database accessible by laboratory geneticists and researchers (e.g. MatchMakerExchange https://www.matchmakerexchange.org/) (26). This may be helpful to gain more insight into the pathogenicity of the variant.*
- *Variant frequencies would ideally be shared publicly so they can be used broadly to facilitate variant filtering and interpretation. Enough information (metadata) should be provided on the type of samples included in the aggregation since the variant frequencies might be biased (e.g., samples from different populations or disease types) and might not be suitable for all scenarios.*

Bioinformatics

- **RECOMMENDATION 15: The use of the most recent annotated reference genome version is recommended.**

  It is preferable to use the latest annotated version of the human reference genome, which should have the highest completeness, curation and accuracy. GRCh38 is a major improvement for CNV and SV detection, but also for SNV detection (27). However, not all content in the patches is diagnostically suitable, so transition to the latest version should be carried out with caution. The established nomenclature of many variants based on previous versions of the reference genome, as well as the lack of annotation sources, might pose a challenge for transitioning to newer genome reference versions.

- **RECOMMENDATION 16: Standard data formats should be used.**

  Initiatives such as the Global Alliance for Genomics and Health (GA4GH) recommend data format standards (https://www.ga4gh.org/).

  Raw reads should be available in FASTQ format so that data can be broadly re-used. Mapped reads should be stored either in BAM or CRAM file format so that alignments can be viewed. SNVs and small indels should be described in gVCF or VCF format. While VCF files only store variant information, gVCF files provide genome wide information: the genotype of each position/region in the genome is documented with quality criteria such as depth and genotype quality. gVCF files are preferably used to compute variant frequencies on a cohort of samples. Other variant types can also be stored in VCF format unless another file format, more suitable than VCF, is advised by the community.

- **RECOMMENDATION 17: The bioinformatics pipeline must be tailored for the technical platform used.**

  For short-read sequencing, the bioinformatics pipeline consists of several tools to get from raw data to annotated variants. First demultiplexing has to be done to assign each sequenced read to the correct biological sample. Then, the reads are mapped to the reference genome and the resulting alignment has to be cleaned prior to variant calling (e.g. removal of PCR and/or optical duplicates, base quality score recalibration). Next, various variant calling tools can be used to detect SNVs, small indels, CNVs, SVs, etc. Finally, the variants are annotated with information from several databases to provide the necessary biological context for variant filtering and interpretation. Some examples of tools commonly used for each step of the analysis are given in supplementary table 2. An alternative to mapping is de novo assembly. This type of analysis may provide better results when looking for SVs and provides haplotype information. However, it remains computationally intense.

  It is recommended to develop the pipeline in a modular fashion, since this will make keeping the pipeline up-to-date easier (i.e. feasible to validate/verify per module).

- **RECOMMENDATION 18: It is recommended to develop and define a protocol to keep the bioinformatics tools used for variant calling and variant annotation up to date.**

  Variant calling and annotation tools should be updated and verified regularly.

  For each module, tool or annotation resource, the version used should be described and traceable for each analysis. Since the tools differ per laboratory no general recommendation is presented here. However, reanalysis of a cohort of (solved) cases should be carried out every time the pipeline/variant interpretation tool is updated to ensure that the variants are still detected and correctly annotated.

- **RECOMMENDATION 19: Optimally characterized reference samples should be used for the validation and standardization of bioinformatics tools.**

The sample type the number of samples to include for validation, and clinically relevant variants/regions should be considered.

  - *Sample type*

Samples that include some commonly encountered variants which have been previously confirmed using an orthogonal laboratory technique, such as the Genome in a Bottle (GIAB, https://www.nist.gov/programs-projects/genome-bottle) should be used. The GIAB consortium not only provides benchmarking germline small variants for a number of cell lines (28) but also tools and procedures for accurately benchmarking both small variants and reference calls (29). The comparison of monozygotic twins and artificial data sets could also be used. Previously characterized samples should also be included to make sure that clinically relevant variants are detected. For example, WES could be compared to WGS of the same samples as part of validation and/or during test optimization.

SV validation is more challenging than small variant validation. However, the GIAB consortium already published a curated SV data set for one cell line (30). The community is actively working on the improvement of SV data sets (31) and the development of tools for SV benchmarking (https://github.com/spiralgenetics/truvari, https://github.com/nhansen/SVanalyzer/blob/master/docs/svbenchmark.rst).

  - *Number of samples/variants*

There is no real consensus on the number of variants to be included in validation studies. Marshall et al. (2020) (32) stated that a low number of samples could be used for SNV and indel validation if those samples are well-accepted reference standards. Using one or two GIAB samples would thus suffice for SNV and small indel validation and would provide better confidence and reliability than only 59 variants, as proposed by (33). For SVs and repeat expansions, a larger number of samples should be used (32).

- *Clinically relevant variants*

New resources are becoming available to ensure that analytically and clinically relevant regions/variants can be accurately detected by the pipeline (34, 35).

- **RECOMMENDATION 20**: **The diagnostic laboratory has to validate all parts of the bioinformatics pipeline (public domain tools or commercial software packages) with standard data sets periodically and whenever relevant changes (new releases) are implemented.**

  The pipeline should be regularly updated so that recent tool versions are used and/or new functionalities can be added. Each pipeline update warrants a new validation/verification. Additionally, whenever changes are made in the wet-laboratory protocol, the (validated) pipeline should be verified to ensure that it can properly handle the new data. If not, a new pipeline release should be made.

- **RECOMMENDATION 21**: **Quality parameters to monitor the analytical process (in process controls) and to measure performance of the used techniques should be adopted. For coding regions, general data quality should be at least similar to that from WES data. All NGS quality metrics used in diagnostics procedures should be accurately described and, ideally, stored in a database.**

  Examples of parameters to monitor for short-read sequencing WGS are provided in supplementary table 3. Observed anomalies may point at wet-laboratory and/or bioinformatics anomalies and should be further investigated. Quality metrics should include thresholds for individual sample data quality such as average depth of coverage, evenness of coverage, percent genome above minimum mapping quality, and/or callability. Samples not meeting minimal quality should either be sequenced again or processed from a newly obtained sample. Note that libraries with a short insert size and/or a high number of duplicates will have

a dramatic impact on the price of WGS. However, if informative coverage is considered (i.e., after exclusion of duplicates, reads of low mapping quality, bases of low Phred quality, and of bases coming from the same DNA fragment) a WGS sample could still pass quality control if enough data has been generated (see supplementary information 2).

- **RECOMMENDATION 22: The bioinformatics pipeline should be validated for all reportable types of variants, minimally including SNVs, small indels, and CNVs.**

  If WGS is implemented to replace WES, the performance of WGS should then be similar to or higher than the one of WES for SNVs and small indels (32). Because the resolution of CNV calling from WGS is expected to outperform WES, CNV calling should also be implemented and validated, replacing array CGH or other methodologies.

  To improve the diagnostic yield of WGS, it is advised to also implement and validate bioinformatics tools detecting:

  - *Aneuploidy and UniParental Disomy (UPD)*
  - *Variants in the mitochondrial genome*
  - *Other (balanced) SVs*
  - *Repeat expansions.*

  The advance in bioinformatics tools may also allow a better detection of variants in paralogous and homologous sequences in the near future. Also, although WGS allows the detection of mosaic variants, a higher depth of coverage than the traditional 30-40X should be used to reliably detect mosaic variants. The detection of mosaic variants would also require a specific validation stating what is the minimal allele frequency at which somatic variants can be called.

  Analytical sensitivity and precision must be established separately for each type of variant during pipeline validation. Usually, analytical sensitivity and specificity are considered. However, given the large number of true negative SNVs (i.e. reference calls) in WGS, it is preferred to focus on sensitivity and precision.

- **RECOMMENDATION 23: All WGS variants should be annotated.**

  Annotation is defined as collecting as much information as possible for the detected variants by using informatics platforms (e.g., DECIPHER, ClinVar, HGMD). Such annotation allows the contextualization of variants by comparison with background variation and variants identified in affected individuals.

  Functional annotation of non-coding regions is still lagging behind that of protein-coding genes. However, population frequency information can easily be retrieved from e.g. gnomAD and/or from in house databases. Ideally, also information on regulatory regions should be available.

- **RECOMMENDATION 24: It is recommended to record variant frequencies in an in-house database.**

  Given the large number of SNVs and CNVs detected from WGS, (local) population frequencies are required to filter out common variants. The use of local variant frequencies may also allow the filtering out of technical artefacts.

- **RECOMMENDATION 25: The diagnostic laboratory should implement a protocol for long-term storage of all relevant data sets.**

  Storage of data is often country specific and no general guideline can be given here. For example, in The Netherlands a distinction is made between temporary files and final results (https://vkgl.nl/nl/kwaliteit/retention-periods). FASTQ and BAM/CRAM files are considered temporary files and should be kept for one year while VCF files are final results and should be kept for five years. DNA should be stored for at least 30 years. In other countries, legal requirements or professional guidelines may apply and/or be available.

Quality assessment

- **RECOMMENDATION 26: The reportable range, that is, the portion of the clinical target for which reliable calls can be generated, has to be defined during the test development and should be available to the clinician.**

  For the detection of SNVs, a mean coverage of at least 30X should suffice in GIAB confident regions. A higher coverage will increase SNV reportable range (see supplementary information 2). While the reportable range can easily be defined at the level of single nucleotides, it is more difficult to apply it for more complex variant types. The list of regions and variant types that cannot be assessed should however be available. For example, common CNVs would probably not be reported if CNVs are detected based on depth of coverage comparison of one sample to a pool of samples or if they are filtered out prior to interpretation. Similarly, current algorithms cannot reliably detect CNVs in, among others, segmental duplications, telomeres, genes with paralogues and/or orthologues. The resolution at which CNVs can be called should be reported to the clinician, as determined during test validation and the testing process.

- **RECOMMENDATION 27: If DNA from different tissue types (*e.g.*, blood and saliva) is tested diagnostically, each tissue type should be validated separately for both wet and dry laboratory procedures.**

  Cell/tissue type and library preparation should be taken into account and included in the validation report (36). The number of raw reads needed to get the appropriate depth of coverage may differ for different tissue types, due to, for example, the difficulty of obtaining fragments with large insert size from certain tissue types.

- **RECOMMENDATION 28: Whenever major changes are made to the test, quality parameters have to be checked, and a set of validation samples has to be re-run as part of the**

validation.

The laboratory should define beforehand the number of cases that have to be assayed whenever the method is updated or upgraded (see also recommendation 19).

- **RECOMMENDATION 29: Aspects of sample tracking and the installation of barcoding to identify samples should be dealt with during the evaluation of the assay and included in the platform validation.**

  Sample swaps can in many cases be identified by analysis of the genotypic data content from a WGS test by checking gender of the sample and the sample relation if several samples from one family are analyzed.

- **RECOMMENDATION 30: Variants compliant with predefined quality metrics do not require confirmation by a second technique.**

  Variant quality scores may be used to identify high confidence variant genotypes for which confirmation by a second technique is not necessary (37).

  However, variants for which no validation has been performed (yet) should be confirmed by an alternative method. For example, repeat expansion could be detected without a validated pipeline but only findings confirmed by an orthogonal method should be reported. This also implies that for conditions in which repeat expansions have to be tested, the WGS test should be complemented with repeat expansion test(s) (cf. diagnostic routing).

  Depending on local policies, confirmation is always necessary for e.g. results with clinical implications such as available treatments depending on a particular genotype (personalized therapies), presymptomatic results, etc.

Ethical considerations

- **RECOMMENDATION 31: Laboratories should have a clearly defined protocol for addressing unsolicited findings prior to launching the test.**

  The odds of detecting UFs greatly depends on the diagnostic strategy. Although laboratories often do not know the exact frequency of detecting UFs, they should at least provide information to requesting physicians on potential outcomes depending on the type of request. E.g., trio analysis focusing on the identification of de novo variants, or a bioinformatics analysis of a restricted gene panel, significantly reduces the odds of UFs. NB: It is essential to provide information on the content of gene panels, since these could harbor genes involved in diseases not relevant to the clinical phenotype (e.g., the ATM gene is involved in breast cancer and ataxia, and the GJB2 gene is involved in deafness and skin disease).

  The local policy about dissemination of UFs should be clear for the requesting physician. This policy should be discussed with the patient in the pre-test counselling to obtain the appropriate consent. The policy should address categories of UFs that can be disclosed, e.g., medically actionable diseases, late onset untreatable conditions, diseases detected in children, or carrier status of a recessive disease, etc.

- **RECOMMENDATION 32: Clinicians should provide genetic counseling and obtain informed consent prior to clinical WGS.**

  Counseling should include discussion of the limitations of testing, likelihood and implications of diagnosis and UFs, and the potential need for further analysis to facilitate clinical interpretation, including studies performed in a research setting (see recommendation 33) (38). Such genetic counselling needs to be done by a qualified clinical expert, such as a clinical geneticist or a medical specialist with specific training in genetic counselling. It is recommended to provide written or online information for patients.

- **RECOMMENDATION 33: The laboratory should anticipate possible follow up studies resulting from the dissemination of unsolicited findings.**

  *Disclosure of UFs will likely result in follow up studies in the family. Since this involves screening of genes beyond the initial diagnostic request, the laboratory might not be able to provide this service and referral to another laboratory is required. Specifically, when the UF protocol includes possible disclosure of carrier status of rare genetic diseases, one has to prepare to receive a request for testing (the entire coding region of the corresponding gene) of partners in order to estimate the risk of affected offspring***.**

- **RECOMMENDATION 34**: **The laboratory is not expected to re-analyze data systematically and report novel findings, unless explicitly requested to do so or for quality assurance activity.**

  Requests for re-analysis of the sequencing data should only be initiated upon a novel diagnostic request (initiated by the referring clinician), or when the patient consented for further analysis of the sequencing data for research purposes (38). The added value of re-analysis should be communicated to the patients i.e., it increases their chance of a molecular diagnosis (39). However, if the laboratory learns that the status of a specific variant has been reclassified (re-interpretation) from a pathogenic or likely pathogenic to a benign or likely benign variant, or vice versa, it is good clinical practice for laboratories to identify patients with this variant and issue a new report to the referring clinician (40). It is therefore obligatory to store data in such a way that those variants/patients can easily be retrieved (see recommendation 13). Thus, reanalysis should be triggered by the referring physician. Patients should be aware and have agreed to this reanalysis. It is currently based on a pull – prescribed by an external health care professional - not a push by the diagnostic laboratory, as the (bio-)informatic tools are

generally not available and the costs are not covered. Nevertheless, reanalysis of existing data is essential for quality assurance activities (e.g. validation of a new pipeline).

- **RECOMMENDATION 35: The results of a diagnostic test, particularly by analysis of a whole genome, might not be conclusive but may be hypothesis generating.**

  The information on the pathogenicity of variants identified in WGS will become available when more laboratories perform diagnostic WGS. Variants with uncertain pathogenicity can be reported as a result of a diagnostic test, because follow up studies might provide more insight in the causality of the variant.

- **RECOMMENDATION 36: WGS data can only be used for research purposes with adequate informed consent.**

  A research test is hypothesis driven and the outcome may have limited clinical relevance for a patient enrolled in the project. When the GDPR came into effect, it became difficult to completely anonymize WGS data (41). Patient data can only be used for research purposes with an appropriate legal basis (e.g. specific consent for the particular project).

Reporting

- **RECOMMENDATION 37: For each NGS test, the laboratory has to provide the following: the diagnostic strategy, the types of genetic variants detected, their reportable range, the analytical sensitivity and precision.**

  To avoid laboratory reports being too long, this information can be provided by linking to a relevant database maintained and updated by the laboratory. This issue was covered in detail in the previous guidelines (42).

- **RECOMMENDATION 38: The report of an NGS assay should summarize the patient's identification and reason for referral, a brief description of the test, a summary of results, and the major findings on one page.**

  Potentially relevant SNVs, CNVs, indels, short tandem repeats, long runs of homozygosity involving a single chromosome (indicative for UPD) or multiple chromosomes (due to consanguinity of the parents) and SVs in a single patient should all be reported in a single report with overarching clinical interpretation.

  The description of the test should include the type of variants that have been analyzed. The tools and databases (and their version) used for analysis (see recommendation 18) should be referred to in the report.

- **RECOMMENDATION 39: Both the reference genome build and, when applicable, the gene reference transcript version should be mentioned in each report.**

- **RECOMMENDATION 40: An OMIM reference should be reported where available (https://www.omim.org/).**

  Online Mendelian Inheritance in Man® (OMIM) is a freely available, comprehensive, and reliable database of human genes and genetic phenotypes. It is advised to refer to this database in the report. However, OMIM is not always up-to-date and sometimes a suitable scientific paper or another database (like Orphanet) may be much more relevant for a certain finding.

- **RECOMMENDATION 41: A local policy, in line with international recommendations, for reporting genomic variants should be established and documented by the laboratory prior to providing analysis of this type.**

Before analyzing WGS data, the laboratory should implement a policy outlining what type of variants will be reported. Important categories to decide upon are carrier status of recessive disease, VUS, and susceptibility variants.

- **RECOMMENDATION 42: VUS should be reported only if the phenotype associated with the respective (disease) gene matches with the clinical features of the patient and when follow up studies can be performed to gain more information about pathogenicity of the variant.**

  Guidelines on the reporting of VUS should take clinical characteristics into account. The report of a VUS should include suggestions for further steps to be taken, i.e., RNA sequencing, functional analysis or segregation in the family. Since trio sequencing already includes segregation analysis, a de novo VUS matching the clinical phenotype can be reported without additional follow up studies.

- **RECOMMENDATION 43: Exploratory findings that are beyond the confirmation or exclusion of a clinical diagnosis should be reported.**

  The identification of variant(s) in a gene not previously associated with a genetic condition can generate reasoning for further research. In order to progress it is recommended that these variants are reported. Variants in candidate genes can also be classified and reported as VUS. For reasons of clarity, a separate report could be issued for such findings.

- **RECOMMENDATION 44: WGS reports should be delivered to the referring physician. Advice to refer the patient and family for genetic counselling must be included in the report.**

  In general, results of genomic testing should reach the referring clinician as soon as possible. Since the outcome of the test can have major implications for other family members, the patient and family should be referred for genetic counselling, especially if prenatal diagnosis

or presymptomatic testing are considered. This is not different for the practice for other genetic tests (43).

References

1. Weiss MM, Van der Zwaag B, Jongbloed JD, Vogel MJ, Bruggenwirth HT, Lekanne Deprez RH, et al. Best practice guidelines for the use of next-generation sequencing applications in genome diagnostics: a national collaborative study of Dutch genome diagnostic laboratories. Hum Mutat. 2013;34(10):1313-21.

2. Bertoli-Avella AM, Beetz C, Ameziane N, Rocha ME, Guatibonza P, Pereira C, et al. Successful application of genome sequencing in a diagnostic setting: 1007 index cases from a clinically heterogeneous cohort. Eur J Hum Genet. 2020.

3. Xue Y, Ankala A, Wilcox WR, Hegde MR. Solving the molecular diagnostic testing conundrum for Mendelian disorders in the era of next-generation sequencing: single-gene, gene panel, or exome/genome sequencing. Genet Med. 2015;17(6):444-51.

4. Lelieveld SH, Spielmann M, Mundlos S, Veltman JA, Gilissen C. Comparison of Exome and Genome Sequencing Technologies for the Complete Capture of Protein-Coding Regions. Hum Mutat. 2015;36(8):815-22.

5. Lionel AC, Costain G, Monfared N, Walker S, Reuter MS, Hosseini SM, et al. Improved diagnostic yield compared with targeted gene sequencing panels suggests a role for whole-genome sequencing as a first-tier genetic test. Genet Med. 2018;20(4):435-43.

6. Lindstrand A, Eisfeldt J, Pettersson M, Carvalho CMB, Kvarnung M, Grigelioniene G, et al. From cytogenetics to cytogenomics: whole-genome sequencing as a first-line test comprehensively captures the diverse spectrum of disease-causing genetic variation underlying intellectual disability. Genome Med. 2019;11(1):68.

7. Dolzhenko E, van Vugt J, Shaw RJ, Bekritsky MA, van Blitterswijk M, Narzisi G, et al. Detection of long repeat expansions from PCR-free whole-genome sequence data. Genome Res. 2017;27(11):1895-903.

8. Genetics ESoH. The European Society of Human Genetics condemns move to impose obligatory genetic testing for employees in the USA 2017 [updated March 16, 2017. Available from: https://www.eshg.org/.

9. Miller DT, Lee K, Chung WK, Gordon AS, Herman GE, Klein TE, et al. ACMG SF v3.0 list for reporting of secondary findings in clinical exome and genome sequencing: a policy statement of the American College of Medical Genetics and Genomics (ACMG). Genet Med. 2021;23(8):1381-90.

10. Fellner A, Ruhrman-Shahar N, Orenstein N, Lidzbarsky G, Shuldiner AR, Gonzaga-Jauregui C, et al. The role of phenotype-based search approaches using public online databases in diagnostics of Mendelian disorders. Genet Med. 2021;23(6):1095-100.

11. Richards S, Aziz N, Bale S, Bick D, Das S, Gastier-Foster J, et al. Standards and guidelines for the interpretation of sequence variants: a joint consensus recommendation of the American College of Medical Genetics and Genomics and the Association for Molecular Pathology. Genet Med. 2015;17(5):405-24.

12. Houge G, Laner A, Cirak S, de Leeuw N, Scheffer H, den Dunnen JT. Stepwise ABC system for classification of any type of genetic variant. Eur J Hum Genet. 2021.

13. ACGS Best Practice Guidelines for Variant Classification in Rare Disease 2020.

14. Liehr T. Cytogenetic contribution to uniparental disomy (UPD). Mol Cytogenet. 2010;3:8.

15. Basel-Salmon L, Orenstein N, Markus-Bustani K, Ruhrman-Shahar N, Kilim Y, Magal N, et al. Improved diagnostics by exome sequencing following raw data reevaluation by clinical geneticists involved in the medical care of the individuals tested. Genet Med. 2019;21(6):1443-51.

16. de Leeuw N, Dijkhuizen T, Hehir-Kwa JY, Carter NP, Feuk L, Firth HV, et al. Diagnostic interpretation of array data using public databases and internet sources. Hum Mutat. 2012;33(6):930-40.

17. Nowakowska B. Clinical interpretation of copy number variants in the human genome. J Appl Genet. 2017;58(4):449-57.

18. Riggs ER, Andersen EF, Cherry AM, Kantarci S, Kearney H, Patel A, et al. Technical standards for the interpretation and reporting of constitutional copy-number variants: a joint consensus recommendation of the American College of Medical Genetics and Genomics (ACMG) and the Clinical Genome Resource (ClinGen). Genet Med. 2020;22(2):245-57.

19. Hochstenbach R, Liehr T, Hastings RJ. Chromosomes in the genomic age. Preserving cytogenomic competence of diagnostic genome laboratories. Eur J Hum Genet. 2021;29(4):541-52.

20. Basel-Salmon L, Ruhrman-Shahar N, Orenstein N, Goldberg Y, Gonzaga-Jauregui C, Shuldiner AR, et al. When phenotype does not match genotype: importance of "real-time" refining of phenotypic information for exome data interpretation. Genet Med. 2021;23(1):215-21.

21. Wright CF, Ware JS, Lucassen AM, Hall A, Middleton A, Rahman N, et al. Genomic variant sharing: a position statement. Wellcome Open Res. 2019;4:22.

22. Telenti A, Jiang X. Treating medical data as a durable asset. Nat Genet. 2020;52(10):1005-10.

23. Ardeshirdavani A, Souche E, Dehaspe L, Van Houdt J, Vermeesch JR, Moreau Y. NGS-Logistics: federated analysis of NGS sequence variants across multiple locations. Genome Med. 2014;6(9):71.

24. van der Velde KJ, Imhann F, Charbon B, Pang C, van Enckevort D, Slofstra M, et al. MOLGENIS research: advanced bioinformatics data software for non-bioinformaticians. Bioinformatics. 2019;35(6):1076-8.

25. Bean LJ, Hegde MR. Gene Variant Databases and Sharing: Creating a Global Genomic Variant Database for Personalized Medicine. Hum Mutat. 2016;37(6):559-63.

26. Philippakis AA, Azzariti DR, Beltran S, Brookes AJ, Brownstein CA, Brudno M, et al. The Matchmaker Exchange: a platform for rare disease gene discovery. Hum Mutat. 2015;36(10):915-21.

27. Li H, Dawood M, Khayat MM, Farek JR, Jhangiani SN, Khan ZM, et al. Exome variant discrepancies due to reference-genome differences. Am J Hum Genet. 2021;108(7):1239-50.

28. Krusche P, Trigg L, Boutros PC, Mason CE, De La Vega FM, Moore BL, et al. Best practices for benchmarking germline small-variant calls in human genomes. Nat Biotechnol. 2019;37(5):555-60.

29. Zook JM, McDaniel J, Olson ND, Wagner J, Parikh H, Heaton H, et al. An open resource for accurately benchmarking small variant and reference calls. Nat Biotechnol. 2019;37(5):561-6.

30. Zook JM, Hansen NF, Olson ND, Chapman L, Mullikin JC, Xiao C, et al. A robust benchmark for detection of germline large deletions and insertions. Nat Biotechnol. 2020;38(11):1347-55.

31. Chapman LM, Spies N, Pai P, Lim CS, Carroll A, Narzisi G, et al. A crowdsourced set of curated structural variants for the human genome. PLoS Comput Biol. 2020;16(6):e1007933.

32. Marshall CR, Chowdhury S, Taft RJ, Lebo MS, Buchan JG, Harrison SM, et al. Best practices for the analytical validation of clinical whole-genome sequencing intended for the diagnosis of germline disease. NPJ Genom Med. 2020;5:47.

33. Roy S, Coldren C, Karunamurthy A, Kip NS, Klee EW, Lincoln SE, et al. Standards and Guidelines for Validating Next-Generation Sequencing Bioinformatics Pipelines: A Joint Recommendation of the Association for Molecular Pathology and the College of American Pathologists. J Mol Diagn. 2018;20(1):4-27.

34. Wagner J, Olson ND, Harris L, McDaniel J, Cheng H, Fungtammasan A, et al. Curated variation benchmarks for challenging medically relevant autosomal genes. Nat Biotechnol. 2022.

35. Wilcox E, Harrison SM, Lockhart E, Voelkerding K, Lubin IM, ClinGen Expert P, et al. Creation of an Expert Curated Variant List for Clinical Genomic Test Development and Validation: A ClinGen and GeT-RM Collaborative Project. J Mol Diagn. 2021;23(11):1500-5.

36. Seth-Smith HMB, Bonfiglio F, Cuenod A, Reist J, Egli A, Wuthrich D. Evaluation of Rapid Library Preparation Protocols for Whole Genome Sequencing Based Outbreak Investigation. Front Public Health. 2019;7:241.

37. Bauer P, Kandaswamy KK, Weiss MER, Paknia O, Werber M, Bertoli-Avella AM, et al. Development of an evidence-based algorithm that optimizes sensitivity and specificity in ES-based diagnostics of a clinically heterogeneous patient population. Genet Med. 2019;21(1):53-61.

38. Boycott K, Hartley T, Adam S, Bernier F, Chong K, Fernandez BA, et al. The clinical application of genome-wide sequencing for monogenic diseases in Canada: Position Statement of the Canadian College of Medical Geneticists. J Med Genet. 2015;52(7):431-7.

39. Liu P, Meng L, Normand EA, Xia F, Song X, Ghazi A, et al. Reanalysis of Clinical Exome Sequencing Data. N Engl J Med. 2019;380(25):2478-80.

40. Vears DF, Senecal K, Clarke AJ, Jackson L, Laberge AM, Lovrecic L, et al. Points to consider for laboratories reporting results from diagnostic genomic sequencing. Eur J Hum Genet. 2018;26(1):36-43.

41. Shabani M, Marelli L. Re-identifiability of genomic data and the GDPR: Assessing the re-identifiability of genomic data in light of the EU General Data Protection Regulation. EMBO Rep. 2019;20(6).

42. Matthijs G, Souche E, Alders M, Corveleyn A, Eck S, Feenstra I, et al. Guidelines for diagnostic next-generation sequencing. Eur J Hum Genet. 2016;24(1):2-5.

43. Claustres M, Kozich V, Dequeker E, Fowler B, Hehir-Kwa JY, Miller K, et al. Recommendations for reporting results of diagnostic genetic testing (biochemical, cytogenetic and molecular genetic). Eur J Hum Genet. 2014;22(2):160-70.

Supplementary table 1: WGS statements and link to WES statements

**Number WGS**	**WGS recommendations (this paper)**	**Number and category in WES guidelines (**Matthijs, Souche *et al.* 2016)
General statements		
1	It is recommended to introduce WGS analysis in a diagnostic setting when it is a relevant improvement on quality, efficiency and/or diagnostic yield.	4, Diagnostic/ clinical utility
2	Diagnostic WGS for rare diseases and cancer (as well as other genetic testing approaches) should only be performed in accredited laboratories.	1, Intro; 35, Distinction between research and diagnostics
3	NGS should not be transferred to clinical practice without acceptable validation of the tests.	1, Intro
4	Confirmation, interpretation and communication to the patient of results obtained in a research setting should always be done after re-testing on (preferably) an independent sample by a diagnostic laboratory.	36, Distinction between research and diagnostics
Diagnostic strategy		
5	The laboratory should provide information to the clinician for which type of variants the genetic test is validated.	2 & 3, Diagnostic/ clinical utility
6	Limitations of WGS should be considered and communicated to the referring clinician.	
7	For diagnostic purposes only genes for which a clear association with the disease has been confirmed, should be reported. Variants in genes of unknown function may be listed in an independent research report.	5, Diagnostic/ clinical utility
8	Diagnostic testing should be directed towards answering the clinical question. It is recommended to preferably analyze one (or more) *in silico* gene panels and use filtering strategies, and, use trios for disorders frequently caused by *de novo* variants.	6, Diagnostic/ clinical utility; 9, 32, Informed consent and information to the patient and clinician; 24, Validation
9	For the interpretation of variants in genes causing a monogenic disorder the '5 tier classification system' should be used.	
10	Large CNVs should be interpreted using databases including cytogenomic aberrations.	
11	It is recommended to analyze and report variants outside the exome only when they are (likely) pathogenic. VUS shall (only) be reported in case follow up studies can provide more insight into pathogenicity.	
12	For interpretation of the variants, it is necessary to have clinical information of the patient (and the parents when trio analysis is performed), preferably in standardized terms, such as HPO.	

13	The diagnostic laboratory has to implement/use a structured database for all classified variants with current annotations.	21, Validation; 31, Reporting
14	Reported variants should be shared by submitting them to federated, regional, national, and/or international databases, accessible by laboratory geneticists and researchers.	38, Distinction between research and diagnostics
Bioinformatics		
15	The use of the most recent annotated reference genome version is recommended.	
16	Standard data formats should be used.	
17	The bioinformatics pipeline must be tailored for the technical platform used.	18, Validation
18	It is recommended to develop and define a protocol to keep the bioinformatics tools used for variant calling and variant annotation up to date.	
19	Optimally characterized reference samples should be used for the validation and standardization of bioinformatics tools.	
20	The diagnostic laboratory has to validate all parts of the bioinformatics pipeline (public domain tools or commercial software packages) with standard data sets periodically and whenever relevant changes (new releases) are implemented.	20, Validation
21	Quality parameters to monitor the analytical process (in process controls) and to measure performance of the used techniques should be adopted. For coding regions, general data quality should be at least similar to that from WES data. All NGS quality metrics used in diagnostics procedures should be accurately described and, ideally, stored in a database.	14 & 15, Validation
22	The bioinformatics pipeline should be validated for all reportable types of variants, minimally including SNVs, small indels, and CNVs.	19, Validation; 17, Validation
23	All WGS variants should be annotated.	
24	It is recommended to record variant frequencies in an in-house database.	37, Distinction between research and diagnostics
25	The diagnostic laboratory should implement a protocol for long-term storage of all relevant data sets.	22, Validation
Quality assessment		
26	The reportable range, that is, the portion of the clinical target for which reliable calls can be generated, has to be defined during the test development and should be available to the clinician.	23, Validation
27	If DNA from different tissue types (*e.g.*, blood and saliva) is tested diagnostically, each tissue type should be validated separately for both wet and dry laboratory procedures.	

28	Whenever major changes are made to the test, quality parameters have to be checked, and a set of validation samples has to be re-run as part of the validation.	25, Validation
29	Aspects of sample tracking and the installation of barcoding to identify samples should be dealt with during the evaluation of the assay and included in the platform validation.	16, Validation
30	Variants compliant with predefined quality metrics do not require confirmation by a second technique.	
Ethical considerations		
31	Laboratories should have a clearly defined protocol for addressing unsolicited findings prior to launching the test.	10, Informed consent and information to the patient and clinician; 29, Reporting; 12, Informed consent and information to the patient and clinician
32	Clinicians should provide genetic counseling and obtain informed consent prior to clinical WGS.	13, Informed consent and information to the patient and clinician
33	The laboratory should anticipate possible follow up studies resulting from the dissemination of unsolicited findings.	11, Informed consent and information to the patient and clinician
34	The laboratory is not expected to re-analyze data systematically and report novel findings, unless explicitly requested to do so or for quality assurance activity.	30, Reporting
35	The results of a diagnostic test, particularly by analysis of a whole genome, might not be conclusive but may be hypothesis generating.	34, Distinction between research and diagnostics
36	WGS data can only be used for research purposes with adequate informed consent.	33, Distinction between research and diagnostics
Reporting		
37	For each NGS test, the laboratory has to provide the following: the diagnostic strategy, the types of genetic variants detected, their reportable range, the analytical sensitivity and precision.	8, Informed consent and information to the patient and clinician
38	The report of an NGS assay should summarize the patient's identification and reason for referral, a brief description of the test, a summary of results, and the major findings on one page.	26, Reporting
39	Both the reference genome build and, when applicable, the gene reference transcript version should be mentioned in each report.	
40	An OMIM reference should be reported where available.	
41	A local policy, in line with international recommendations, for reporting genomic variants should be established and documented by the laboratory prior to providing analysis of this type.	27, Reporting

42	VUS should be reported only if the phenotype associated with the respective (disease) gene matches with the clinical features of the patient and when follow up studies can be performed to gain more information about pathogenicity of the variant.	28, Distinction between research and diagnostics
43	Exploratory findings that are beyond the confirmation or exclusion of a clinical diagnosis should be reported.	
44	WGS reports should be delivered to the referring physician. Advice to refer the patient and family for genetic counselling must be included in the report.	

Supplementary table 2: List of WGS processing steps, bioinformatics tools, databases and file formats

Processing step	Description	Tools and databases	Output
**Base calling and demultiplexing**	Base calling and demultiplexing, are also referred as primary analysis.	vendor software of the sequencing platform	FASTQ file(s)
**Adapter trimming (optional)**	Sequencing adapters may be trimmed from the read ends for those reads where the insert size is smaller than the read length. If not trimmed and/or not properly handled by the mapping tool, sequenced adapters may interfere with mapping and variant calling, leading to false-positive or false-negative variant.	CutAdapt [1], BWA [2](soft clipping while mapping), Trimmomatic [3], SeqPrep [4], SeqPurge [5]	FASTQ files or BAM file (if soft clipping by a mapper such as BWA)
**Low-quality trimming (optional)**	Low quality bases may also interfere with mapping and variant calling and can be trimmed from the end (and begin) of reads.	CutAdapt [1], BWA [2] (soft clipping while mapping), Trimmomatic [3], SeqPrep [4]	FASTQ files or BAM file (if soft clipping by a mapper such as BWA)
**Mapping**	In the read mapping step, paired-end/ single-end reads are mapped to the reference genome allowing for base changes and indels. Mapping should always be performed against the full reference genome even when a small gene panel is analysed.	BWA [2], Novalign [6], Stampy [7], SOAP2 [8], Bowtie [9]	BAM file
**Duplicate removal (optional)**	In shotgun sequencing few duplicates are expected since the DNA is randomly sheared. However, duplicates can occur during PCR and as an artifact of imaging.	Picard MarkDuplicates [10], SAMBLASTER [11]	BAM file
**Indel realignment (optional)**	Local realignment around indels may improve indel calling accuracy.	ABRA2 [12]	BAM file
**Quality score recalibration (optional)**	After mapping to the reference genome, the base quality score of the reads can be recalibrated to better match the probability of false base calls and to spread the quality scores wider over the valid range. In most algorithms, false base calls are distinguished from real variants by performing a simple base calling or using databases of known polymorphisms.	GATK BaseRecalibrator & PrintReads [13], ReQON [14]	BAM file
**BAM file quality check**	BAM file quality can be assessed by varioustools in order to infer the informative coverage and check whether the sample passes QC.	Picard CollectWGSMetrics [10], MosDepth[15]	TSV, TXT files
**SNV calling**	SNV calling consists of detecting and genotyping differences to the reference genome (base changes and small indels).	GATK HaplotypeCaller [13], samtools [16], FreeBayes [17], DeepVariant [18], Platypus [19]	VCF file

Processing step	Description	Tools and databases	Output
**CNV calling**	CNV calling consists of detecting and genotyping CNVs. CNV can either be called by comparing the deprh of coverage of one sample to the depth of coverge of a set of refernece samples, in which case common CNVs will not be detected or by using read pair or split read information, inwhich case all CNVs can be detected. CNV calling remains difficult in repetive regions using short read data.	CNVnator [20], Control-FREEC [21], ClinCNV [22], Manta [23], Lumpy [24]	VCF, TSV, TXT files
**SV calling**	SV calling consists of detecting and genotyping SVs. SVs can either be called using read pair information, split read information or assembly. SV calling remains difficult in repetive regions using short read data.	Manta [23], Lumpy [24], BreakDancer [25]	VCF, TSV, TXT files
**Repeat expansion calling**	Repeat expansion calling consists of inferring the number of repeats of STRs. This analysis can either be limited to STRs known to cause disease or assess the number of repeats of all STRs larger than in the referene genome (*de novo* STR detection).	ExpansionHunter [26], STRetch [27]	
**Annotation**	Variant interpretation requires detailed annotation. Very basic annotations are gene name, region (exonic, splicing, intronic, intergenic, etc.) and coding change information. Additionally, minor allele frequency for known polymorphisms, pathogenicity and conservation scores and clinical databases can be used.	Annovar [28], SNPeff [29], VEP [30], Agilent Alissa Interpret [31], dbSNP [32], 1000 Genomes [33], GnomAD [34], SIFT [35], PhyloP [36], MutationTaster [37], COSMIC [38], OMIM [39], ClinVar [40], HGMD [41]	CSV, TSV, TXT, excel files or databases
**Filtering**	To find disease related variants in large variant lists, rigorous filtering is needed. Typical variant filters exclude low quality variants, synonymous SNPs or known polymorphisms with high frequencies in the population. However, this kind of filtering selects both for deleterious and false-positive variant calls. To remove the false-positives, filtering according to variant frequencies of an *in-house* database, containing all the processed samples of a lab, is often applied. Because an *in-house* database accumulates false-positive variants that are specific for the used sequencing platform, sequencer and analysis pipeline, it can be used to identify and remove these false-positives.	Agilent Alissa Interpret [31], SnpSift [42]	CSV, TSV, TXT, excel files or databases

References

1. Martin M. Cutadapt removes adapter sequences from high-throughput sequencing reads. EMBnet.journal 2011; 17:10-12.
2. Li H, Durbin R. Fast and accurate short read alignment with Burrows-Wheeler Transform. Bioinformatics 2009; 25:1754-60.
3. Bolger AM, Lohse M, Usadel B. Trimmomatic: a flexible trimmer for Illumina sequence data; Bioinformatics 2014; 30:2114-20.
4. John St. J. SeqPrep: Tool for stripping adaptors and/or merging paired reads with overlap into single reads.2011. https://github.com/jstjohn/SeqPrep. Accessed 15 December 2021.
5. Sturm M, Schroeder C, Bauer P. SeqPurge: highly-sensitive adapter trimming for paired-end NGS data. BMC Bioinformatics 2016; 17:208.
6. Novocraft Technologies. Novalign: Powerful tool designed for mapping of short reads onto a reference genome from Illumina, Ion Torrent, and 454 NGS platforms. 2014. http://www.novocraft.com/products/novoalign. Accessed 15 December 2021.
7. Lunter G, Goodson M. Stampy: a statistical algorithm for sensitive and fast mapping of Illumina sequence reads. Genome Res 2011; 21:936-939.
8. Li R, Yu C, Li Y, Lam T-W, Yiu S-M, Kristiansen K et al. SOAP2: an improved ultrafast tool for short read alignment. Bioinformatics 2009; 25:1966-1967.
9. Langmead B, Salzberg S. Fast gapped-read alignment with Bowtie 2. Nat Methods 2012; 9:357-359.
10. Broad institute. Picard tools. 2009. https://broadinstitute.github.io/picard/. Accessed 15 December 2021.
11. Faust GG, Hall IM. SAMBLASTER: fast duplicate marking and structural variant read extraction. Bioinformatics 2014. 30:2503–2505.
12. Mose LE, Perou CM, Parker JS. Improved indel detection in DNA and RNA via realignment with ABRA2. Bioinformatics 2019; 35:2966–2973.
13. DePristo M, Banks E, Poplin R, Garimella KV, Maguire JR, Hartl C et al. A framework for variation discovery and genotyping using next-generation DNA sequencing data. Nat Genet 2011; 43:491-498.
14. Cabanski CR, Cavin K, Bizon C, Wilkerson MD, Parker JS, Wilhelmsen KC et al. ReQON: a Bioconductor package for recalibrating quality scores from next-generation sequencing data. BMC Bioinformatics 2012; 13:221.
15. Pedersen BS, Quinlan AR. Mosdepth: quick coverage calculation for genomes and exomes. Bioinformatics 2018; 34:867-868.
16. Li H, Handsaker B, Wysoker A, Fennell T, Ruan J, Homer N et al. 1000 genome project data processing subgroup; the Sequence Alignment/Map format and SAMtools. Bioinformatics 2009;25:2078-2079.
17. Garrison E, Marth G. Haplotype-based variant detection from short-read sequencing. arXiv preprint arXiv:1207.3907 [q-bio.GN] 2012.
18. Poplin R, Chang P-C, Alexander D, Schwartz S, Colthurst T, Ku A, et al. A universal SNP and small-indel variant caller using deep neural networks. Nature Biotechnology 2018; 36:983–987.
19. Rimmer A, Phan H, Mathieson I, Iqbal Z, Twigg SRF, WGS500 Consortium, et al. Integrating mapping-, assembly- and haplotype-based approaches for calling variants in clinical sequencing applications. Nat Genet 2014; 46:912–918.
20. Abyzov A, Urban AE, Snyder M, Gerstein M. CNVnator: an approach to discover, genotype, and characterize typical and atypical CNVs from family and population genome sequencing. Genome Res 2011; 21:974-84.
21. Boeva V, Popova T, Bleakley K, Chiche P, Cappo J, Schleiermacher G, et al. Control-FREEC: a tool for assessing copy number and allelic content using next generation sequencing data. Bioinformatics 2012; 28:423-5.
22.Demidov G, Ossowski S. ClinCNV: novel method for allele-specific somatic copy-number alterations detection. 2019. BioRxiv
23. Chen X, Schulz-Trieglaff O, Shaw R, Barnes B, Schlesinger F, Källberg M et al. Manta: rapid detection of structural variants and indels for germline and cancer sequencing applications. Bioinformatics 2016; 32:1220-2.

24. Layer RM, Chiang C, Quinlan AR, Hall IM. LUMPY: a probabilistic framework for structural variant discovery. Genome Biology 2014; 15:R84.
25. Chen K, Wallis JW, McLellan MD, Larson DE, Kalicki JM, Pohl CS et al. BreakDancer: An algorithm for high resolution mapping of genomic structural variation. Nat Methods 2009; 6:677–681.
26. Dolzhenko E, Deshpande V, Schlesinger F, Krusche P, Petrovski R, Chen S et al. ExpansionHunter: a sequence-graph-based tool to analyze variation in short tandem repeat regions. Bioinformatics 2019; 35:4754–4756.
27. Dashnow H, Lek M, Phipson B, Halman A, Sadedin S, Lonsdale A et al. STRetch: detecting and discovering pathogenic short tandem repeat expansions. Genome Biology 2018; 19:121.
28. Wang K, Li M, Hakonarson H. ANNOVAR: Functional annotation of genetic variants from next-generation sequencing data. Nucleic Acids Res 2010; 38:e164.
29. Cingolani P, Platts A, Wang LL, Coon M, Nguyen T, Wang L et al. A program for annotating and predicting the effects of single nucleotide polymorphisms, SnpEff: SNPs in the genome of Drosophila melanogaster strain w1118; iso-2;iso-3. Fly 2012; 6:80-92.
30. McLaren W, Gil L, Hunt SE, et al. The Ensembl Variant Effect Predictor. Genome Biology 2016; 17:122.
31. Agilent Alissa Interpret. https://www.agilent.com/en/product/next-generation-sequencing/clinical-informatics-platform/alissa-interpret-930086. Accessed 15 December 2021.
32. Sherry ST, Ward MH, Kholodov M, Baker J, Phan L, Smigielski EM et al. dbSNP: the NCBI database of genetic variation. Nucleic Acids Res 2001; 29:308-311.
33. The 1000 genomes project consortium. An integrated map of genetic variation from 1,092 human genomes. Nature 2012; 491:56–65.
34. Karczewski KJ, Francioli LC, Tiao G, Cummings BB, Alföldi J, Wang Q et al. The mutational constraint spectrum quantified from variation in 141,456 humans. Nature 2020; 581:434–443.
35. Kumar P, Henikoff S, Ng PC. Predicting the effects of coding non-synonymous variants on protein function using the SIFT algorithm. Nat Protoc 2009; 4:1073-1081.
36. Cooper GM, Stone EA, Asimenos G, NISC Comparative Sequencing Program, Green ED, Batzoglou S et al. Distribution and intensity of constraint in mammalian genomic sequence. Genome Res 2005; 15:901-913.
37. Schwarz JM, Rödelsperger C, Schuelke M, Seelow D. MutationTaster evaluates disease-causing potential of sequence alterations. Nat Methods 2010; 7:575–576.
38. Tate JG, Bamford S, Jubb HC, Sondka Z, Beare DM, Bindal N et al. The Catalogue of Somatic Mutations in Cancer (COSMIC). Nucleic Acids Res 2019;47(D1):D941-D947.
39. McKusick-Nathans Institute of Genetic Medicine, Johns Hopkins University (Baltimore, MD). Online Mendelian Inheritance in Man, OMIM®. 1996. http://omim.org/. Accessed 15 December 2021.
40. Landrum MJ, Lee JM, Riley GR, Jang W, Rubinstein WS, Church DM et al. ClinVar: public archive of relationships among sequence variation and human phenotype. Nucl Acids Res 2014; 42:D980-D985.
41. Stenson PD, Mort M, Ball EV, Shaw K, Phillips A, Cooper DN. The Human Gene Mutation Database: building a comprehensive mutation repository for clinical and molecular genetics, diagnostic testing and personalized genomic medicine. Hum Genet 2014; 133:1-9.
42. Cingolani P, Patel VM, Coon M, Nguyen T, Land SJ, Ruden DM et al. Using Drosophila melanogaster as a model for genotoxic chemical mutational studies with a new program, SnpSift. Front Genet 2012; 15:3-35.

Supplementary table 3: Metrics that can be used for WGS quality control

Quality metrics based on raw reads (FASTQ) or mapped reads (BAM)	
Parameter	***Comment***
median base quality by cycle	Base quality typically decreases towards the end of the reads. As a rule of thumb, the quality score should not fall below 20 (Phred quality score).
percentage of bases with Q30	The percentage of bases with Q30 indicates whether the run succeeded or not. This percentage should meet the vendor criteria.
percentage duplicate reads or percentage of bases excluded because of duplicate	The percentage of duplicate reads is an indicator of the library complexity.
percentage trimmed bases (if applicable)	The percentage of trimmed bases during adapter trimming.
percentage of mapped reads	The percentage of reads that could be mapped to the reference genome.
average informative coverage	The average sequencing depth across the genome, after removal of duplicates, overlapping bases, bases of low quality,
standard deviation of informative coverage	The standard deviation of informative coverage of the genome, *i.e.* after all filters are applied. This metric can be used to assess coverage uniformity.
percentage of bases excluded because of low mapping quality	The percentage of aligned bases excluded because of low mapping quality (below 20).
percentage of bases excluded because of low base quality	The percentage of aligned bases excluded because of low base quality (below 20).
percentage of bases excluded because mate read is not mapped	The fraction of aligned bases excluded because they were in reads without a mapped mate pair.
percentage of bases excluded because of overalp (in case of paired-end sequencing)	The percentage of bases excluded because of overlap asseses the insert size of the sequence library. The shorter the insert size, the more bases will be excluded because of overlap. Indeed for short fragments, one part of the fragment will be sequenced in both R1 and R2 reads. However, only one copy should be retained in variant calling for a better estimate of variant frequencies. The variant caller should be able to exclude overlapping bases. Those bases should also b exlcuded from informative coverage calculation. A high proportion of overlapping bases should not prevent a sample to pass QC if the cut-off on informative coverage is met. It will nontheless have a direct impact on the cost of WGS (as more reads will ave to be sequenced to meet the informative coverage criteria).
percentage of bases excluded because of too high coverage	The percentage of aligned bases excluded because of too high coverage. This metric can be used to assess coverage uniformity.
percentage of target region with depth 20 or more	The percentage of the genome sequenced with an informative depth greater than or equal to 20 (or any other informative depth considered to be the minimum for diagnostics).

Quality metrics based on SNV (VCF)	
Parameter	***Comment***
total number of variants	The total number of variants should be similar for samples which were processed with the same pipeline and have the same ethnicity.
percentage of variants known polymorphisms	Most detected variants (> 90%) of each sample should be known polymorphisms. The proportion of known polymorphisms depends on the sample's ethnicity as well as the ethnicity of samples used to generate the known polymorphisms.
percentage of variants indels	The percentage of indels with respect to the total number of variants.
percentage of variants homozygous	The percentage of homozygous variants with respect to the total number of variants.
percentage of nonsense variants	The percentage of nonsense variants with respect to the total number of variants.
transition/transversion ratio	The ratio of transitions/transversions.

Quality metrics based on CNV	
Parameter	***Comment***
total number of variants	The total number of variants should be similar for samples which were processed with the same pipeline and have the same ethnicity.

Supplementary information 2: Impact of library quality and coverage on sensitivity and precision.

To assess the impact of library quality and coverage on sensitivity and precision, two different libraries made for cell line GM12878 have been compared.

The first library (later referred to as “library not optimized”) was not fully optimized: the fragments were too short, causing 9% of the sequenced bases to be discarded from variant calling and mean informative coverage calculation. Additionally, more than 20% of the bases were discarded from the analysis as duplicates. These duplicates come either from the library or a suboptimal sample loading on the sequencer. In total, more than 35% of sequenced bases were excluded from the analysis. Although it is still possible to reach a mean informative coverage of 39X by resequencing, the number of reads to sequence to reach such a coverage is very high (more than 1.2 million reads, Figure 1).

For the second library (later referred as “optimized library”), only 17% of the sequenced bases were excluded from the analysis. This sample has initially been sequenced at a mean coverage of 49X and subsequently down-sampled to mean informative coverage of 25X, 30X and 42X. A mean informative coverage of 42X can be obtained with one million reads (Figure 1).

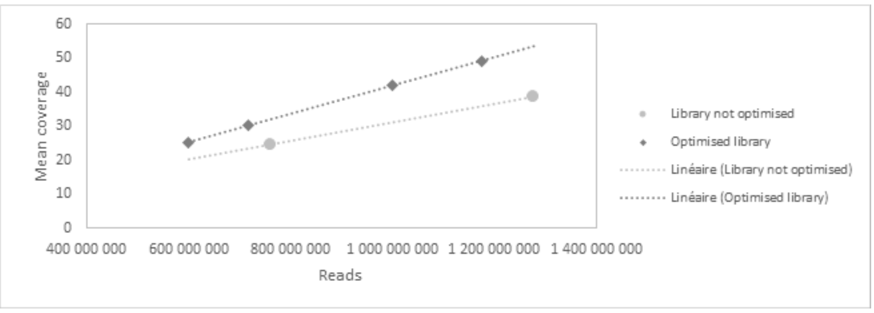

Figure 1. Mean informative coverage by number of sequenced reads for two different libraries. The library not optimized has a larger number of duplicates and shorter insert size than the optimized library.

Despite the lower library quality, the proportion of the genome covered by 1, 5, 10, 15, 20, 25, 30, 40, 50, 60, 70, 80, 90 and 100X is very similar for both libraries when sequenced at the same mean informative coverage (Figure 2, mean coverage of 25X).

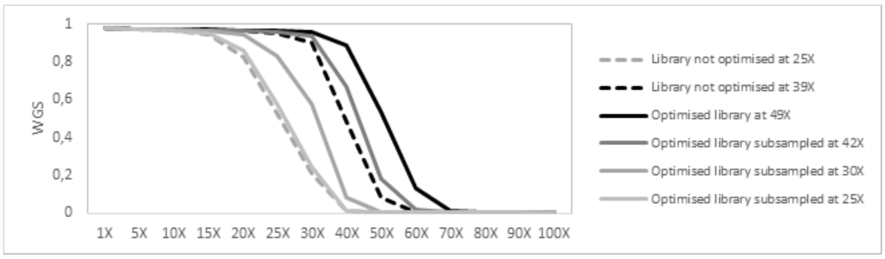

Figure 2. Proportion of WGS covered by 1, 2, etc. reads for optimized and not optimized libraries sequenced at different mean informative coverage.

The reportable range for SNVs is very similar for both libraries (Figure 3). Precision and sensitivity of SNV calling are also very similar for both libraries, being above 0.9935 and 0.9993, respectively. Down-sampling to a lower mean informative coverage does not have a major impact on precision and sensitivity (Figure 3). The number of called GIAB variants decreases with the mean coverage from 3,037,553 (98.5% of GIAB variants) at 49X to 3,029,285 (98.2% of GIAB variants) at 25X. However, the variants that are no longer detected when decreasing the informative coverage do not pass QC and are therefore not considered as false negatives as they are not part of the reportable range.

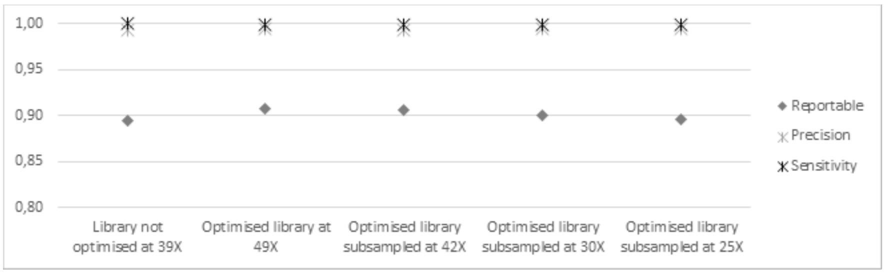

Figure 3. Proportion of reportable WGS, precision and sensitivity for optimized and not optimized libraries sequenced at different mean informative coverage.
